# The Uncommon Suspect: Pseudomonas aeruginosa and Cavitary Lung Lesions in an Immunocompetent Patient

**DOI:** 10.7759/cureus.66075

**Published:** 2024-08-03

**Authors:** Nishant Allena, Mahnoor Arshad, Zoraize Moeez Athar, Srikaran Bojja, Ravish Singhal

**Affiliations:** 1 Pulmonary Medicine, BronxCare Health System, New York, USA; 2 Internal Medicine, BronxCare Health System, New York, USA; 3 Internal Medicine, BronxCare Health System, Icahn School of Medicine at Mount Sinai, New York, USA; 4 Pulmonary and Critical Care Medicine, BronxCare Health System, New York, USA

**Keywords:** pulmonary cavitation, lung cavitation, upper lobe cavitary lesion, bronchoscopy, levofloxacin, aztreonam, bronchoalveolar lavage (bal), cavitary lung lesion, pseudomonas aeruginosa (p. aeruginosa), community-acquired pneumonia (cap)

## Abstract

Cavitary lung lesions pose a formidable diagnostic challenge due to their multifaceted etiologies. While tuberculosis and other prevalent pathogens typically dominate discussions, instances of community-acquired *Pseudomonas aeruginosa* (*P. aeruginosa*) pneumonia leading to cavitation in immunocompetent individuals remain exceptionally rare. Herein, we present a compelling case of such pneumonia in a 61-year-old man with a past medical history of hypertension and coronary artery disease who presented with cough, chest pain, and subjective fever. Chest imaging revealed cavitary lung lesions, which is atypical for community-acquired pneumonia (CAP). Initial workup excluded common CAP pathogens, following which bronchoscopy with bronchoalveolar lavage (BAL) definitively diagnosed *P. aeruginosa*, prompting targeted antibiotic therapy. Treatment led to clinical and radiographic improvement. *P. aeruginosa* rarely causes CAP, especially in immunocompetent patients, and cavitary lesions further complicate diagnosis. This case highlights the importance of considering *P. aeruginosa* in CAP with unusual features and emphasizes the utility of bronchoscopy with BAL for diagnosis and guiding management.

## Introduction

Cavitary lung lesions can arise from various clinical conditions, such as infectious diseases, and autoimmune and malignancies. Organisms such as *Mycobacterium tuberculosis*, *Staphylococcus aureus*, *Streptococcus pneumoniae*, and *Klebsiella pneumoniae* are commonly identified pathogens. However, community-acquired pneumonia (CAP) manifesting as a cavitary lung lesion is uncommon, with *P. aeruginosa* even more rarely reported as the causative organism. This case report details an immunocompetent individual with CAP caused by *P. aeruginosa* presenting as a cavitary lung lesion, highlighting the role of bronchoscopy with bronchoalveolar lavage (BAL) in the diagnosis and management of such cases.

## Case presentation

Our patient is a 61-year-old male, who has a past medical history of hypertension, coronary artery disease (with a history of myocardial infarction and subsequent percutaneous coronary intervention involving placement of two stents), and asthma. The patient presented to the emergency room with chest pain and a persistent cough, which started one week before presentation. The cough was predominantly dry but occasionally productive with yellowish sputum. Additionally, he reported symptoms of nasal congestion, sore throat, and rhinorrhea for the same duration. A review of symptoms was negative for hemoptysis, leg swelling, or calf pain. Despite the absence of measured fever, he described a subjective fever accompanied by chills and night sweats for two days. 

**Figure 1:**
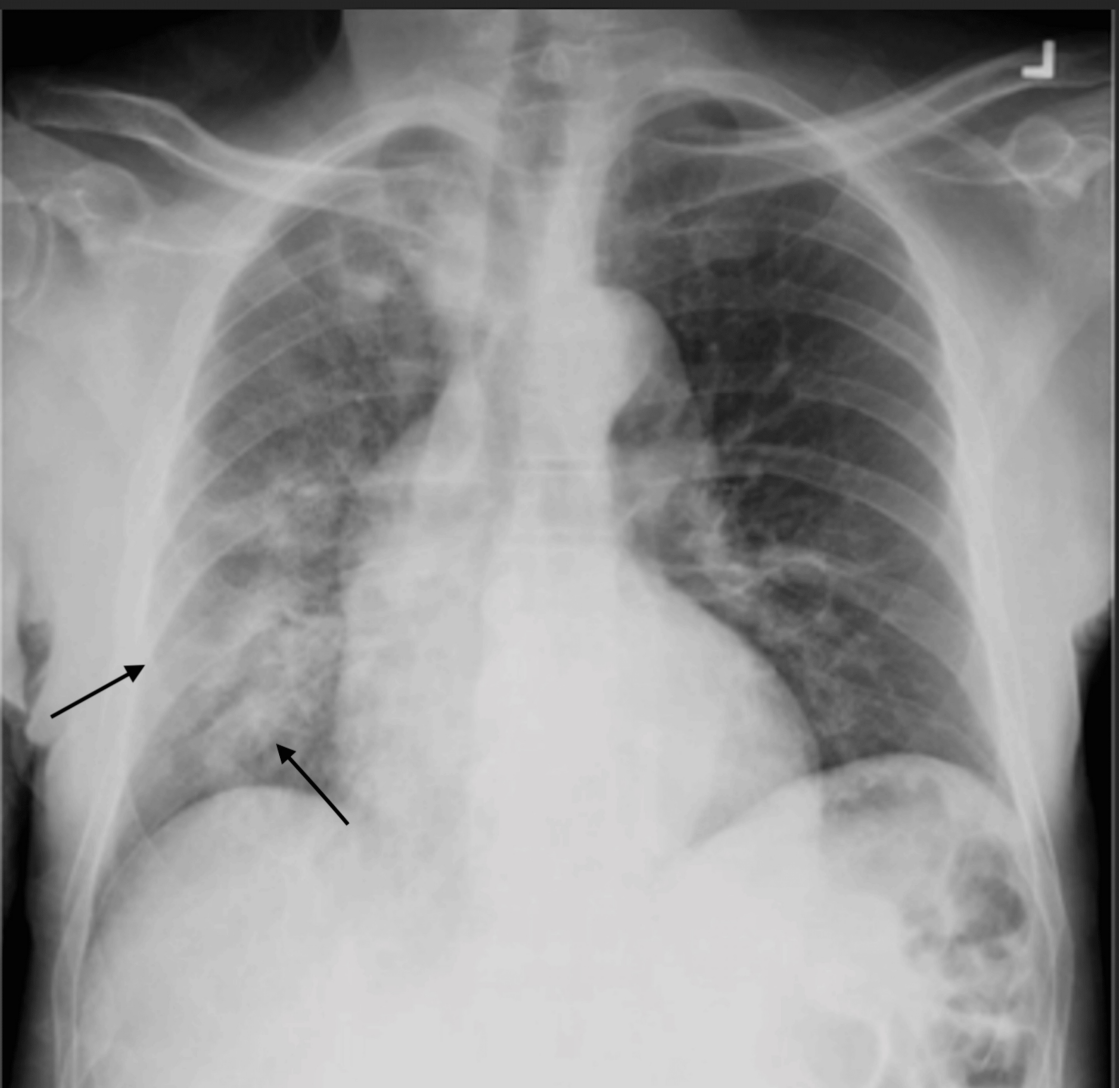
Anterior to posterior view of the chest Diffuse right-sided infiltrates are seen as marked by the black arrows.

His social history was significant for a recent two-month incarceration followed by release four months before admission. He used to smoke and quit 20 years ago, along with social alcohol use. There is also no reported history of substance use. A chest X-ray revealed diffuse right-sided opacities (Figure 1).

Table 1 summarizes the results of pertinent laboratory tests.

**Table 1:** Pertinent results CRP = C-reactive protein, HIV = Human immunodeficiency virus, k/μL = 1000 per microliter, g/dL = gram per deciliter, meq/L = milliequivalent per liter, mg/L = milligram per liter

Test	Result	Normal Range
White Blood Cell Count	19.8 k/μL	4.8-10.8 k/μL
Neutrophil Percentage	89.2%	40-70%
Hemoglobin	11.3 g/dL	12-16 g/dL
Serum Sodium	132 meq/L	135-145 meq/L
CRP	249.24 mg/L	<5 mg/L
HIV antibody	Negative	Negative

**Figure 2:**
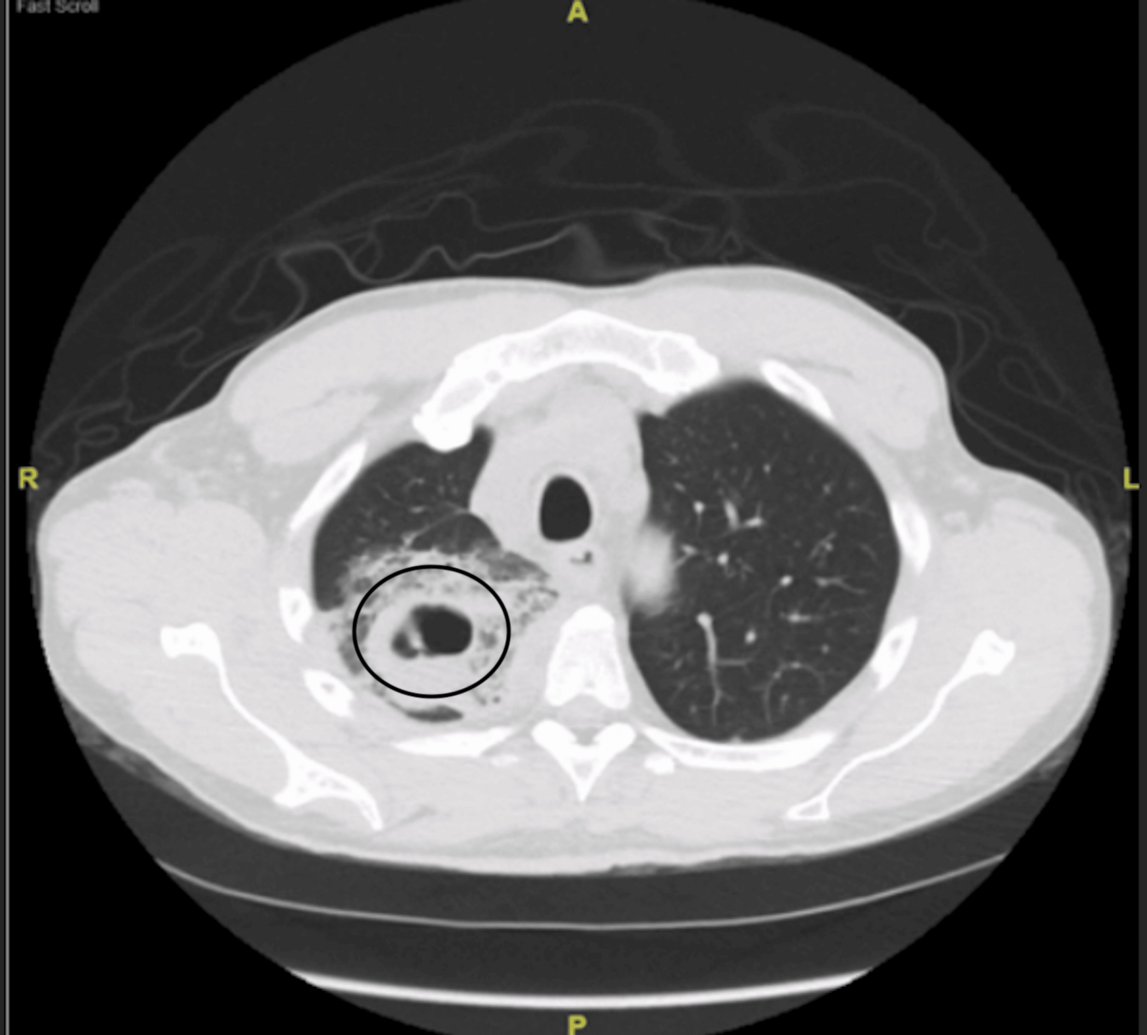
Cavitary lesion seen in the right upper lobe (encircled)

**Figure 3:**
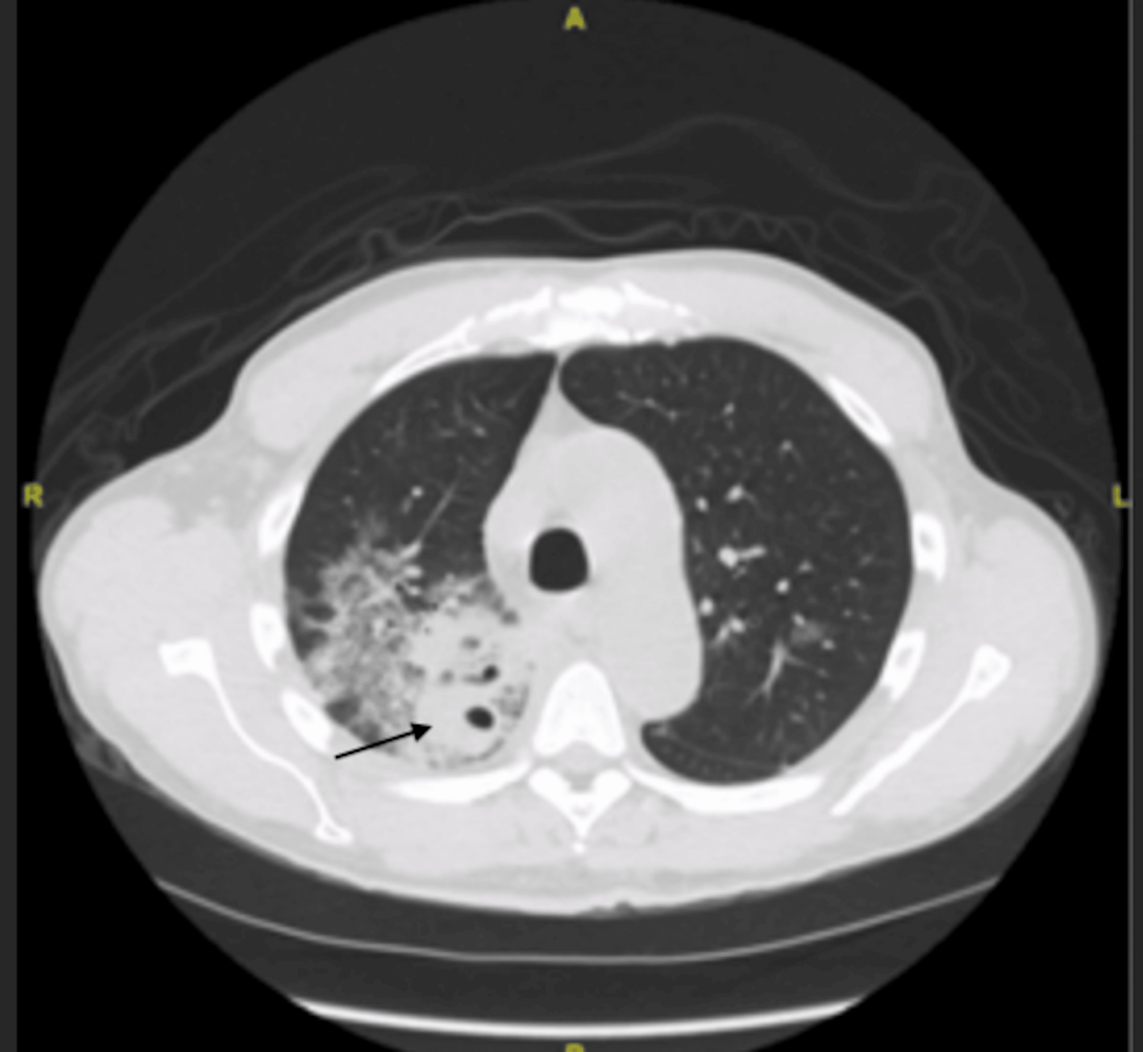
Dense consolidation in the right lower lobe (black arrow)

The patient was admitted to the medical floor and was started on empiric therapy with vancomycin, azithromycin, and aztreonam. The choice of antibiotics was influenced by the fact that the patient had a penicillin allergy. Otherwise, we would have gone with piperacillin-tazobactam instead of aztreonam. Subsequently, a computed tomography (CT) of the chest was performed, revealing multiple cavitary lesions in the right upper lobe (Figure 2), with dense infiltrates in the right lower lobe (Figure 3).

**Figure 4:**
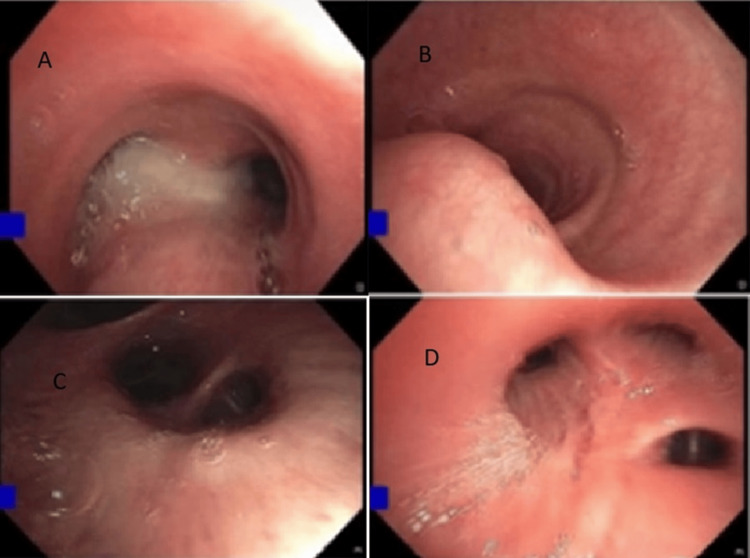
Fiberoptic bronchoscopy images showing mucopurulent secretions in the carina and right upper lobe A = Carina, B = Trachea, C = Right lower lobe, D = Right upper lobe

Sputum samples for acid-fast bacilli (AFB) and culture revealed negative results. A bronchoscopy (Figure 4) with BAL yielded negative results for *M. tuberculosis *and *P. jirovecii *pneumonia (PCP) but was positive for *P. aeruginosa*, which was pan-sensitive. The patient continued aztreonam for a total of four weeks. Vancomycin and azithromycin were not continued after the culture results. 

**Figure 5:**
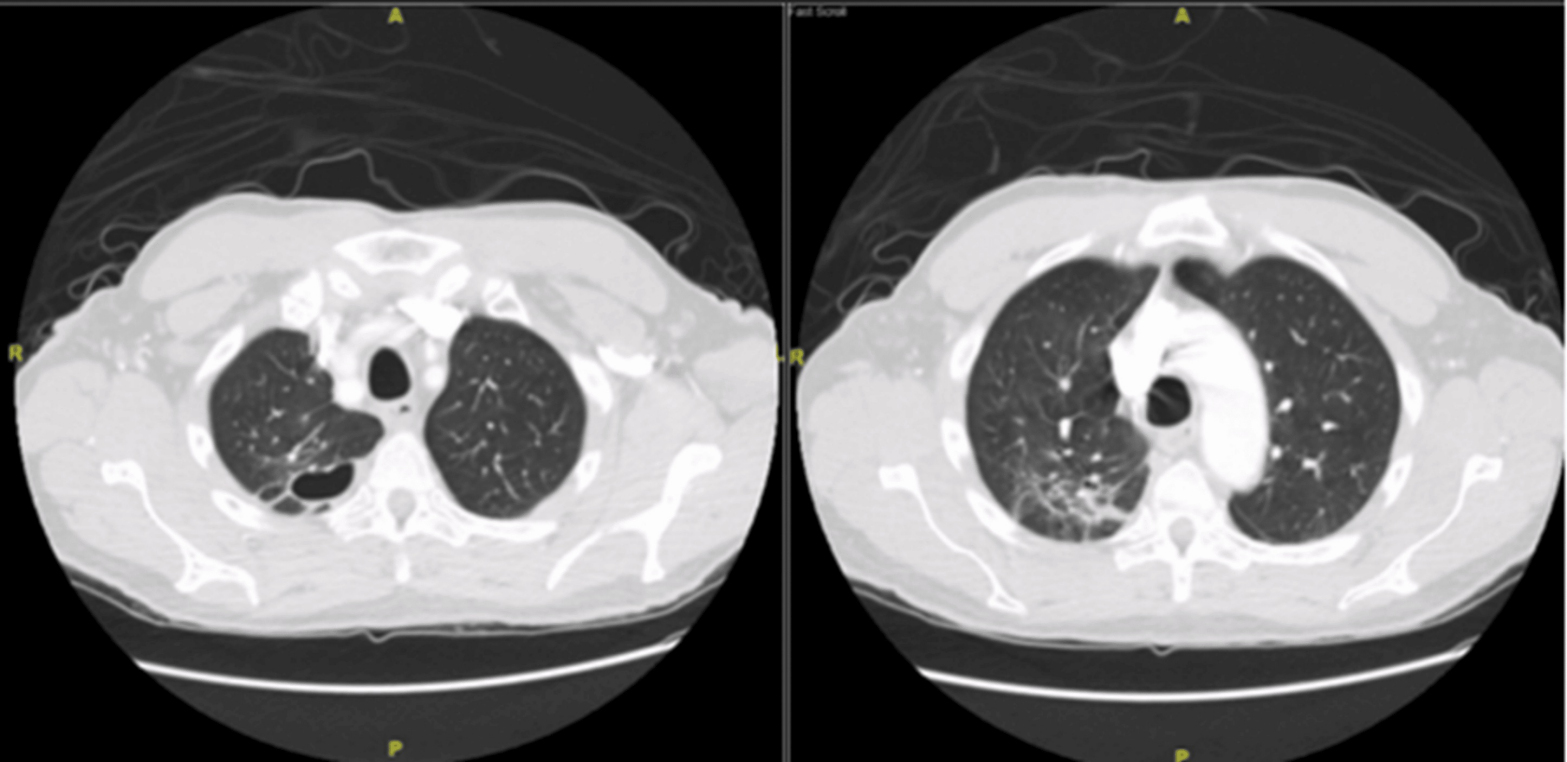
CT chest after completion of treatment Cavitary lesion on the right upper lobe (left image) and reduced consolidation in the right lower lobe (right image)

The patient was discharged from the hospital on oral levofloxacin for an additional four weeks. A CT scan of the chest post-treatment (Figure 5) showed a decrease in the size of the cavitary lesions and resolved pneumonia.

## Discussion

*P. aeruginosa*, an aerobic Gram-negative rod, is a notorious pathogen for causing nosocomial pneumonia but rarely causes CAP in immunocompetent individuals. Most cases occur in patients with underlying risk factors such as lung disease, immunodeficiency, or prolonged antibiotic use. When patients with CAP are displaying unusual rapid growth or treatment-resistant disease courses, *P. aeruginosa* should be considered a causative pathogen [1].

Studies show *P. aeruginosa* CAP prevalence at 4.2% and necrotizing pneumonia complicating 0.8-7% of CAP cases [2]. A review by Hatchette et al. [3] found two-thirds of cases involve the right upper lobe, as seen in our patient. However, a thorough literature review did not yield any findings as to why *Pseudomonas* pneumonia-causing cavitation predominantly involves upper lobes, primarily because the phenomenon is rare and not enough cases have been observed to pinpoint an exact pathophysiology. It is to be noted that various other organisms, such as *S. aureus,* *M. tuberculosis*, *Klebsiella *spp., and *Burkholderia pseudomallei *are also notorious for causing upper lobe lung cavitations [4]. Our hypothesis is that the mechanism may be less related to the causative organism itself and more dependent on the basic physiology of the lung, which tells us that the upper lobes of the lung are well oxygenated and relatively less perfused than lung bases, offering poorer lymphatic drainage in upper lungs, therefore making the region more prone to formation of abscesses and necrosis [5].

*Pseudomonas* CAP can lead to rapid deterioration, acute respiratory failure, and septic shock, with variable treatment durations of two to six weeks based on the clinical course and radiological resolution of the cavity. Table 2 summarizes our review of case reports regarding *Pseudomonas* pneumonia-causing cavitation and their outcomes. It is to be noted the presentations and clinical courses of these cases were widely different ranging from mild symptomatic disease to septic shock requiring vasopressors.

**Table 2:** Case reports where P. aeruginosa was identified as the causative organism and the infection resulted in lung cavitation

Author Name	Location of Cavity	Antibiotic Administered	Outcome
Fujii et al. [6]	Right Upper Lobe	Tazocillin	Clinically Improved
Crnich et al. [7]	Right Upper Lobe	Ciprofloxacin	Clinically Improved
Gharabaghi et al. [8]	Left Upper Lobe	Ciprofloxacin	Clinically Improved
Kunimasa et al. [9]	Right Upper Lobe	Meropenem + Levofloxacin	Clinically Improved
Maharaj et al. [10]	Right Upper Lobe	Ceftazidime	Clinically Improved
Okamoto et al. [11]	Right Upper Lobe	Meropenem + Ciprofloxacin	Deceased
Quirk et al. [12]	Left Upper Lobe	Ceftazidime	Clinically Improved
Rivière et al. [13]	Right Upper Lobe	Cefepime	Clinically Improved
Sakamoto et al. [14]	Left Upper Lobe	Ampicillin/Sulbactam	Deceased
Shaulov et al. [15]	Right Upper Lobe	Ceftriaxone + Azithromycin + Metronidazole	Deceased
Vikram et al. [16]	Right Upper Lobe	Ciprofloxacin	Clinically Improved
Allena et al. (our case)	Right Upper Lobe	Aztreonam + Levofloxacin	Clinically Improved

In our case, monotherapy with aztreonam for four weeks and then levofloxacin for an additional four weeks yielded positive clinical results. In a retrospective cohort study conducted among patients who received either aztreonam or an antipseudomonal beta-lactam as empiric therapy, Hogan et al. found that, in patients with a penicillin allergy, such as the patient discussed in this report, monotherapy with aztreonam is found to have an increased rate of treatment failure compared to the beta-lactam group [17]. They suggested that, despite the risk of hypersensitivity reactions during the use of beta-lactam treatment, there was a significant reduction in clinical failure (p<0.004) [17]. In severe allergy cases, aztreonam was more easily tolerated. It yielded similar 30-day hospital mortality rates, concluding that the use of aztreonam monotherapy should be reserved for those with severe allergic responses since more effective alternative options exist [17].

Flexible bronchoscopy has become a standard of care for diagnosing and treating critically ill patients. In more than 40% of CAP cases, the causative organism remains unidentified, and since proper treatment is crucial, early bronchoscopy can modify and aid in the prognosis of the disease course [18]. BAL and bronchial washing are both safe and minimally invasive bronchoscopic techniques indicated for severe lung diseases which can provide specimens for microbiological examination. With its excellent safety profile with a mortality rate of less than 0.05%, it can be used in critically ill patients while monitoring vital parameters [19].

## Conclusions

Recognition of *P. aeruginosa* in cavitary lesions, albeit infrequent in immunocompetent patients, holds significant clinical importance owing to its potential to cause necrotizing pneumonia. Bronchoscopy serves as a valuable diagnostic modality, particularly in scenarios where conventional sputum cultures yield negative results. Initiation of antibiotic therapy, informed by susceptibility testing to address multidrug resistance concerns, is imperative. Rigorous monitoring, alongside imaging studies, is essential to assess therapeutic efficacy and potential complications.
